# A randomized controlled trial of a proactive analgesic protocol demonstrates reduced opioid use among hospitalized adults with inflammatory bowel disease

**DOI:** 10.1038/s41598-023-48126-0

**Published:** 2023-12-16

**Authors:** Sameer K. Berry, Will Takakura, Devin Patel, Rajalakshmi Govalan, Afsoon Ghafari, Elizabeth Kiefer, Shao-Chi Huang, Catherine Bresee, Teryl K. Nuckols, Gil Y. Melmed

**Affiliations:** 1 Department of Medicine, Cedars Sinai Medical Center, Los Angeles, USA; 2 F. Widjaja Inflammatory Bowel Disease Institute, Karsh Division of Gastroenterology, Department of Medicine, Cedars-Sinai Medical Center, Los Angeles, CA 90048 USA; 3 Research Informatics and Scientific Computing Core, Cedars-Sinai Medical Center, Los Angeles, USA; 4 Biostatistics and Bioinformatics Core, Cedars-Sinai Medical Center, Los Angeles, USA

**Keywords:** Crohn's disease, Ulcerative colitis

## Abstract

Most hospitalized patients with inflammatory bowel disease (IBD) experience pain. Despite the known risks associated with opioids in IBD including risk for misuse, overdose, infection, readmission, and even death, opioid use is more prevalent in IBD than any other chronic gastrointestinal condition. Most hospitalized IBD patients receive opioids; however, opioids have not been shown to improve pain during hospitalization. We conducted a randomized controlled trial in hospitalized patients with IBD to evaluate the impact of a proactive opioid-sparing analgesic protocol. Wearable devices measured activity and sleep throughout their hospitalization. Chronic opioid users, post-operative, and pregnant patients were excluded. The primary endpoint was a change in pain scores from admission to discharge. Secondary endpoints included opioid use, functional activity, sleep duration and quality, and length of stay. Of 329 adults with IBD evaluated for eligibility, 33 were enrolled and randomized to the intervention or usual care. Both the intervention and control group demonstrated significant decreases in pain scores from admission to discharge (− 2.6 ± 2.6 vs. − 3.0 ± 3.2). Those randomized to the intervention tended to have lower pain scores than the control group regardless of hospital day (3.02 ± 0.90 vs. 4.29 ± 0.81, *p* = 0.059), used significantly fewer opioids (daily MME 11.8 ± 15.3 vs. 30.9 ± 42.2, *p* = 0.027), and had a significantly higher step count by Day 4 (2330 ± 1709 vs. 1050 ± 1214; *p* = 0.014). There were no differences in sleep duration, sleep quality, readmission, or length-of-stay between the two groups. A proactive analgesic protocol does not result in worsening pain but does significantly reduce opioid-use in hospitalized IBD patients.

Clinical trial registration number: NCT03798405 (Registered 10/01/2019).

## Introduction

Inflammatory bowel disease (IBD) is a chronic gastrointestinal condition which encompasses both Crohn’s disease (CD) and ulcerative colitis (UC). People with IBD may require hospitalization due to relapsing inflammation and disease complications. Hospitalized patients with IBD experience a variety of symptoms, with the majority experiencing pain^1^. Pain in IBD is rated by patients as one of their most burdensome symptoms, yet there is insufficient evidence to support a specific pain management strategy^2–4^. IBD patients admitted to the hospital with pain are twice as likely to be readmitted within 30 days compared to those with IBD admitted for other issues^5^. Currently, pain in hospitalized patients with IBD is frequently treated with opioids, and often on an as-needed, reactive basis. This reactive approach to pain control often leaves patients in pain for longer than necessary and may theoretically lead to pain escalation and greater use of analgesics. Furthermore, nearly half of patients with IBD are prescribed opioids upon hospital discharge^1^.

Opioid use is one of ten outcome measures used to measure quality of IBD care^6^. Opioid use in IBD is associated with diverse adverse outcomes including an increased risk of opioid overdose, death and infection, increased length-of-stay and readmission, higher healthcare costs, increased emergency room use, and failure to respond to biologic therapy^7–11^. Inpatient opioid use can mask assessment of disease activity due to sedating and constipating side-effects. Despite the risks of opioids in IBD, opioid use continues to be more prevalent in IBD than any other chronic gastrointestinal condition and opioid use disorders among patients with IBD are increasing 8.8% annually^9,12^.

Strategies to reduce opioid-use have been successfully implemented in colorectal surgery with the Enhanced Recovery After Surgery (ERAS) protocol. This is a proactive, multimodal analgesic bundle which has demonstrated reduced opioid-use, improved pain scores, and reduced hospital length-of-stay^13–18^. Similar results have been seen in post-operative IBD patients on the ERAS protocol, but there is limited data on these approaches in non-operative patients hospitalized with IBD^19,20^. IBD patients represent a special population due to the adverse outcomes associated with opioids and a very high rate of ongoing opioid use after discharge.

Given the known risks associated with opioid-use in IBD, any intervention that decreases opioid consumption without negatively impacting pain control is likely to improve the safety of IBD care—but the implications for the effectiveness of pain control are less clear^8,11^. In a retrospective observational analysis, a multi-modal analgesic approach was associated with reduced opioid-use in hospitalized patients with IBD^21^. To better understand the implications of an opioid-sparing approach to pain control we conducted a randomized controlled trial to compare a proactive, opioid-sparing analgesic protocol to usual care among hospitalized patients with IBD.

## Materials and methods

### Study design and population

We conducted a single-center, randomized controlled trial in accordance with CONSORT guidelines comparing a proactive protocol to usual care in adults with IBD in the hospital experiencing pain and admitted for an IBD-related diagnosis including diarrhea, abdominal pain, arthralgias, gastrointestinal bleeding, abdominal abscess, bowel obstruction, or fistula-related complications. Patients who had undergone surgery within the last 30 days or who had a confirmed alternative gastrointestinal diagnosis to account for pain were excluded. Subjects were also excluded if they met any of the following criteria: pregnancy, age < 18, chronic (daily) opioid use prior to admission, admission orders with strict *nil per os* (NPO), or if in the opinion of the treating physician, their pain was clearly unrelated to IBD. This trial was conducted at Cedars-Sinai Medical Center in Los Angeles, California; patient recruitment occurred from January 2019 to March 2020 and was halted early due to research restrictions on non-COVID-19 clinical research instituted due to the coronavirus pandemic. All methods were carried out in accordance with relevant guidelines and regulations and all experimental protocols were approved by the Cedars-Sinai Medical Center Institutional Review Board (IRB). All admitted patients were screened daily for eligibility using a reporting workbench tool in the electronic medical record (EMR), which assessed clinical criteria in real-time to identify potential subjects. Eligible subjects were approached for enrollment within 24 h of hospitalization. Consented patients were randomized to the proactive protocol (intervention arm) or to usual care (control arm) using a web-based in-house randomization software (RANDI2.3), centrally allocated, and with block randomization to ensure equal enrollment between CD and UC^22^.

### Proactive protocol (intervention arm)

**Figure 1:**
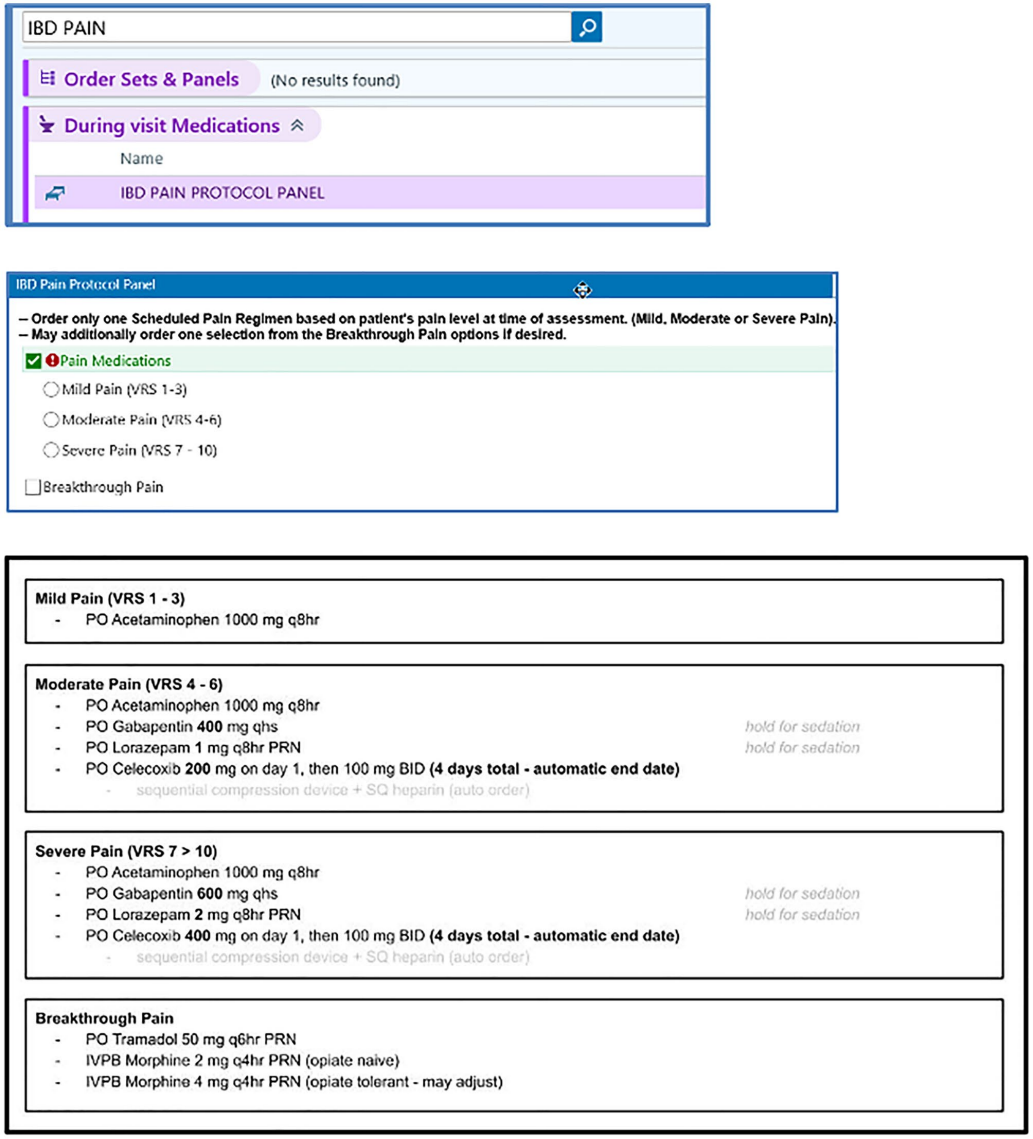
Proactive analgesic protocol and EMR ordering. Proactive analgesic protocol. Patients randomized to the intervention arm were given the above regimen based on their pain score. VRE = Verbal Rating Scale, PO = Per os, IVPB = intravenous piggy-back.

Subjects assigned to the intervention received the Proactive Analgesic Inpatient Narcotic-Sparing (P.A.I.N.-Sparing) protocol (Fig. 1). The protocol included pre-specified medication dose, route, and frequency, with nurse-instructed holding parameters for sedation. The protocol was informed by systematic literature review in discussion with a multidisciplinary panel that included pharmacists, IBD-specialized gastroenterologists, and physicians with expertise in chronic pain management. Prior to implementation in the present study, the protocol was offered to 10 patients who provided additional feedback. The protocol included escalating regimens for mild (1–3), moderate (4–6), and severe (7–10) pain. The P.A.I.N.-Sparing protocol was an integrated order set in the EMR, and the regimen could be modified at the discretion of the primary attending physicians. Orders were automatically discontinued after a certain number of doses. Breakthrough opioid pain medications could be used if non-opioid analgesics failed to adequately control pain, but required additional physician evaluation and were not automatically prescribed.

### Usual care (control arm)

Patients in the control arm received usual care for pain at the discretion of their admitting hospitalist. Typically, this is comprised of a “reactive” sliding scale, where patients are prescribed escalating doses of opioid analgesics for increased pain on the 0–10 numeric pain rating scale (below) as well as as-needed acetaminophen. At our institution, there are standardized inpatient analgesic ordersets which most admitting providers will use. These inpatient ordersets usually consist of 1 mg of morphine or 0.5 mg of hydromorphone administered intravenously every 4–6 h PRN.

### Endpoints

The primary endpoint was a change in patient-reported pain scores on a 11-point numeric rating scale from admission to discharge. Secondary endpoints were opioid use, functional activity including sleep, and healthcare utilization (readmissions, length of stay) between the intervention and control group.

### Assessment of pain

The 11-point numeric rating scale was used to assess pain intensity. This scale has demonstrated validity, test–retest reliability, and responsiveness to changes in pain^23^. Nurses recorded self-reported pain with every vital sign check and at the time of analgesic administration, and then documented the scores in the EMR along with pain location, sedation level, and administered medications. We calculated daily average pain scores based on all recorded observations over each 24-h period, starting at midnight.

### Assessment of opioid use

Opioid use was extracted automatically from the EMR using structured query language (SQL), and converted into morphine milligram equivalents (MME) using standardized formulas to allow for comparison between different opioids^24^. Opioid medications administered for procedural sedation were excluded.

### Assessment of functional activity and sleep

We assessed sleep and physical activity using an activity-tracking wearable device (FitBit Charge 2®). This device uses a variety of sensors to determine sleep duration and quality (deep, light, rapid eye movement (REM)), and step count. Fitbit® data was analyzed using Fitabase®, a remote monitoring tool offered by FitBit® for researchers to monitor, collect, and analyze data for clinical trials in a HIPAA-compliant format.

### Assessment of healthcare utilization and demographics

Healthcare utilization and baseline patient characteristics were extracted from the EMR with SQL. We collected each patient’s length of stay in days, and 30-day readmission rates to our hospital. Demographic data including age, gender, race, and IBD type (CD, UC) were recorded. We also assessed metrics regarding IBD activity and interventions including need for surgery during admission and objective markers of inflammation (C-reactive protein, (CRP)).

### Statistical analysis

Subjects were analyzed per-protocol. Patient characteristics were compared across groups with Student’s t-test (for continuous data) or Fisher’s Exact Test (for categorical data). Longitudinal data was compared across groups over time with mixed model regression with an auto-regressive covariance structure with time modeled as a categorical variable given the non-linear nature of the data. In all cases, residuals were inspected to ensure the fit of the modeling and where necessary, data was Box-Cox transformed (daily MME, sleep time, steps taken) prior to analysis to meet assumptions necessary for parametric testing. Separate modeling was performed to test factors relating to patient characteristics (specific IBD diagnosis, CRP levels). Least-square means estimates were computed for overall differences between groups and over time. Raw Spearman Rank correlations were calculated. In all testing, data were considered significant at the two-tailed *p*-value < 0.05. Data are presented as means with standard deviations (SD) or counts with percentages. Analysis was performed using SAS v9.4 (Carey, NC). Data analysis for Fitbit® use was analyzed from days 1 (Admission date) through discharge or day 7, whichever came first.

### Sample size

We assumed that 70% of usual care subjects would consume opioids, with an average daily pain score of 4.4, a standard deviation of 2.2, and an intra-subject correlation of 0.9 across observations with a change of at least 0.8 points in average daily pain scores over time^1^. Based on simulations (v14)^31^, we estimated 84% power to detect a difference of at least 1-point in average daily pain scores between treatment groups (our primary hypothesis), and 80% power to detect a similar change between the significant covariate of opioid-users and non-users over time with mixed-model regression, two-sided alpha level of 0.05, for a sample size of 83 subjects per group. All relevant interaction terms would have > 90% power to detect a minimal difference of at least 0.5-points. Simulations were run assuming 5 days of data.

This research protocol was approved by our institutional review board. All subjects provided written, informed consent. Informed consent was obtained from all subjects and/or their legal guardian(s). This clinical trial was registered on clinicaltrials.gov (Identifier: NCT03798405 10/01/2019).

## Results

### Baseline characteristics

**Figure 2:**
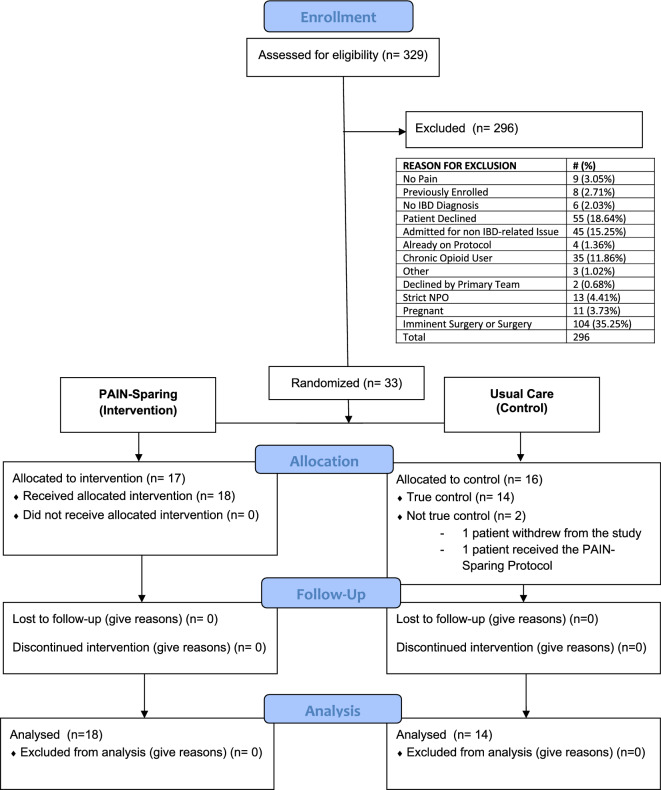
CONSORT flow diagram. Enrollment numbers and reasons for exclusion.

We identified 329 patients with an IBD-related admission during the study period, of whom 88 were eligible; 33 patients enrolled (Fig. 2). Study enrollment was halted early due to a higher-than-expected number of ineligible subjects and COVID-19 research restrictions implemented in March, 2020 that limited non-COVID clinical trial activities. Of the 33 enrolled patients, 1 withdrew consent prior to randomization and was excluded from analysis. Seventeen were randomized to the intervention and fifteen to usual care. One subject randomized to usual care received the proactive protocol. Baseline demographics including ethnicity, age, gender, type of IBD, CRP, and reason for admission were similar between the two groups (Table 1). There were no adverse events in either group attributable to analgesic medications.

**Table 1:** Baseline characteristics.

	Intervention (N = 18)	Control (N = 14)	*p* value
Age, years (SD)	36.9 (9.2)	43.4 (11.4)	0.089^a^
Gender			
Female	11 (61.1%)	9 (64.3%)	> 0.999^b^
Male	7 (38.9%)	5 (35.7%)	
Race			
White	11 (61.1%)	10 (71.4%)	0.529^b^
Asian	3 (16.7%)	0 (0.0%)	
Black	2 (11.1%)	1 (7.1%)	
Hispanic	2 (11.1%)	2 (14.3%)	
Other	0 (0.0%)	1 (7.1%)	
Diagnosis			
CD	9 (50.0%)	9 (64.3%)	0.490^b^
UC	9 (50.0%)	5 (35.7%)	
Diagnosis type			
Flare	15 (83.3%)	11 (78.6%)	0.816^b^
Infection	1 (5.6%)	2 (14.3%)	
Other	2 (11.1%)	1 (7.1%)	
Surgical case	2 (11.1%)	3 (21.4%)	0.631^b^
Hospital readmission < 30d	2 (11.1%)	2 (14.3%)	> 0.999^b^
LOS, days (SD)	7.3 (6.6)	7.1 (3.5)	0.886^a^
CRP at admission, mg/L (SD)	40.3 (44.1)	26.0 (35.4)	0.337^a^

*p*-values computed by (a) Student’s t-test or (b) Fisher’s Exact Test.

SD, standard deviation; CD, Crohn’s disease; UC, Ulcerative colitis; d, day; CRP, C-reactive protein.

### Pain

**Figure 3:**
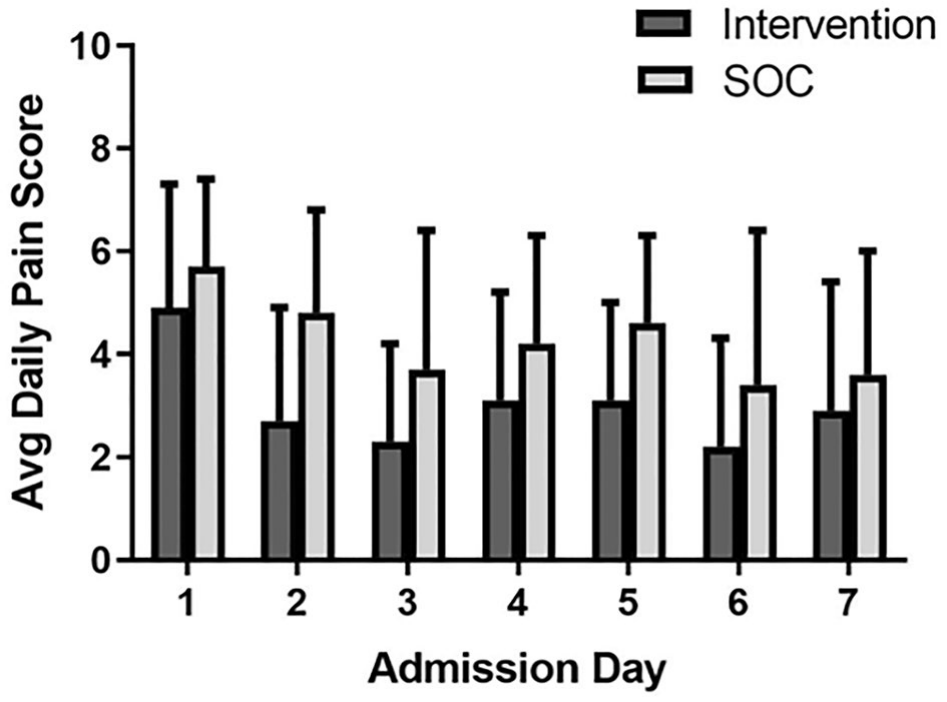
Pain scores. Depicts average daily pain score over time. Pain score was based on a numeric rating scale and scored from 0 to 10. While both groups demonstrated a significant decrease in daily pain scores over time (*p* < 0.001), there was a trend favoring the intervention group with overall lower daily pain scores (3.0 ± 0.9 vs. 4.3 ± 0.8, *p* = 0.059). SOC = standard of care.

There was a similar decrease in pain over time in both groups with an estimated average decrease of 2.8 ± 2.8 points from days 1 to 7 (*p* < 0.001). Overall, those receiving the intervention tended to have lower pain scores than the usual care group regardless of the hospital day (3.02 ± 0.90 vs. 4.29 ± 0.81, *p* = 0.059) (Fig. 3). Pain scores in the usual care group did not improve faster over time when compared to the intervention group. Neither the type of IBD (CD vs. UC) nor CRP value on admission were associated with pain scores.

### Opioid use

**Figure 4:**
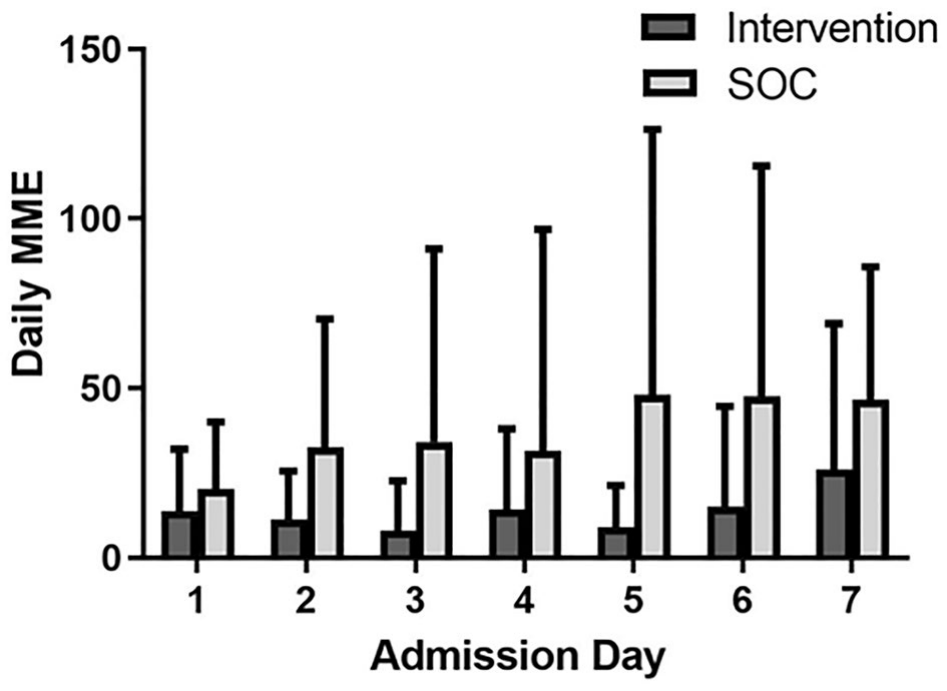
Opioid use. Average daily MME by hospitalization day. Patients in the intervention arm used significantly less MME than patients in the control arm (13.94 ± 5.96 vs. 37.26 ± 10.51, *p* = 0.027). MME = morphine milligram equivalents. SOC = standard of care.

Patients randomized to the intervention consumed significantly fewer opioids than the usual care group (average daily MMEs: 11.8 ± 15.3 vs. 30.9 ± 42.2, *p* = 0.027) (Fig. 4). There was no significant difference in opioid use over time (*p* = 0.573) or with the interaction of treatment group with time (*p* = 0.595). Neither the type of IBD (CD vs. UC) nor CRP value on admission (marker of disease severity) were associated with inpatient opioid-use.

### Sleep and functional activity

**Figure 5:**
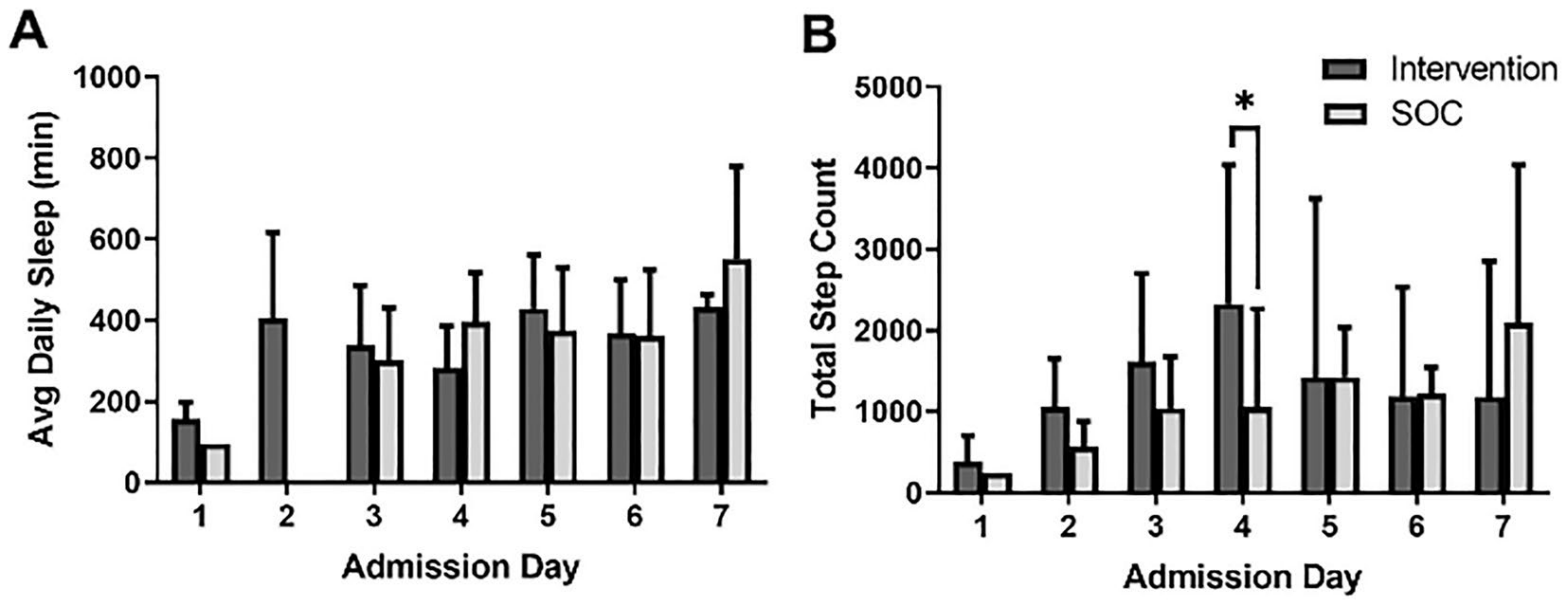
Sleep and functional activity. (**A**) depicts minutes slept and (**B**) depicts steps taken by each subject on their respective hospitalization days where significant differences were observed at Day 4. (**A**) has no value for the control armon day 2 due to Fitbit malfunctioning. SOC = standard of care.

Fifteen subjects in the proactive protocol group and 11 subjects in the usual care group had sleep and activity data. There were no differences in daily sleep duration between the intervention and usual care groups (345 ± 99 vs. 348 ± 148 min, *p* = 0.635), or over time (*p* = 0.133), or by the interaction of group with time (*p* = 0.408). There was a significantly higher cumulative step count by Day 4 in the proactive group than the usual care group (2330 ± 1709 vs. 1050 ± 1214; *p* = 0.014) (Fig. 5). Increased opioid-use (daily MME) was associated with a shorter time spent in deep sleep (r =  − 0.285, *p* = 0.037). There were no associations between daily pain and daily sleep duration, sleep quality, or step count. Neither the type of IBD (CD vs. UC) nor CRP value on admission were associated with sleep duration.

### Healthcare utilization

There were no differences in hospital length-of-stay (7.3 ± 6.6 days vs. 7.1 ± 3.5 days, *p* = 0.66), surgery during admission (11.1% vs. 21.4%, *p* = 0.63), or 30-day readmission rates (11.1% vs. 14.3%, *p* > 0.99) between those assigned to the intervention and those receiving usual care.

## Discussion

Our study has several important findings. First, we found that patients randomized to a Proactive Analgesic Inpatient Narcotic-Sparing (P.A.I.N.-Sparing) protocol consumed significantly less opioids than patients randomized to usual care. Furthermore, patients randomized to the intervention also had numerically lower overall pain scores without an increase in length-of-stay or 30-day readmission rates. Those in the usual care group used more than twice the dose of daily opioids yet did not have improved pain control. These data suggest potential beneficial treatment effects without obvious associated harm.

We also found that patients randomized to the proactive protocol had significantly higher functional activity by hospital day 4 than patients in the usual care group. We hypothesized that some patients may continue to report high pain scores even with pain improvement due to fear of reduced access to analgesic medications, and thus included functional activity assessments in our study as a surrogate marker for pain. Functional activity measured by wearable devices have recently been reported to correlate with disease activity and pain^25^. This improvement in functional activity we identified in those receiving the proactive pain protocol thus further suggests improved pain-control relative to usual care. Increased step count is also known to reduce the risk of deep vein thrombosis (DVT), depression, and anxiety, which are all known complications of patients admitted for IBD flare^26,27^. These exploratory analyses warrant further study.

There is a paucity of published data on the optimal management of pain in hospitalized patients with IBD. A recent observational study looked at the impact of encouraging providers to use a multi-modal, opioid-sparing approach in hospitalized patients with IBD and found a reduction in parenteral opioid consumption and total parenteral opioid dose after implementing their educational program^21^. They were able to demonstrate this reduction in opioid-use by recommending non-opioid analgesics through consultant notes and text messages to providers. While some of the medications in this study were similar to those included in our protocol, there are important differences. We included scheduled, rather than as-needed, analgesics which could include oral acetaminophen, gabapentin, celecoxib, and benzodiazepines before opioids were offered. We were restricted from inclusion of intravenous acetaminophen due to its high cost compared with oral acetaminophen and did not include anti-cholinergics due to the risks of these medications in patients with bowel obstruction, severe ulcerative colitis, or toxic megacolon.

Our study had notable limitations. First, patient recruitment did not achieve target enrollment as we stopped the trial due to the COVID-19 pandemic. Furthermore, a high proportion of subjects screened were ineligible for the study. However, despite a limited sample size, we still detected significant differences in opioid consumption between the two study arms. Second, our protocol only included pharmacologic interventions. Given the known impact of psychological stress on pain in IBD, non-pharmacological interventions such as virtual reality or cognitive-behavioral therapy might further improve opioid use and pain. Third, our study was conducted at a single center with a tertiary care population and may not be generalizable to all populations. Other limitations include that we were unable to categorize pain scores for the control group the same way as the intervention group (escalating regimens for mild (1–3), moderate (4–6), and severe (7–10) pain). It was interesting that 55 of 88 eligible patients declined to participate in the study; reasons for this may include hesitation or anxiety around participating in a study which could limit their access to analgesics. There was also no blinding of study participants as we felt this would additional logistical barriers to the hospitalist teams and nursing staff.

In summary, we found that hospitalized patients with IBD randomized to a proactive analgesic protocol used significantly fewer opioids than usual care, without worsening of pain, length-of-stay, or readmissions. Furthermore, those admitted for IBD flare also had greater functional activity, in the form of a higher daily step count compared to usual care. Future studies will be needed to further validate these findings and explore the impact of other innovative strategies to improve pain during hospitalization for patients with IBD.

## Data Availability

The datasets generated and/or analyzed during the current study are not publicly available due lack of patient consent and institutional restrictions on public data sharing, but are available from the corresponding author on reasonable request.
